# One health clones of multidrug-resistant *Escherichia coli* carried by synanthropic animals in Brazil

**DOI:** 10.1016/j.onehlt.2022.100476

**Published:** 2022-12-21

**Authors:** Elder Sano, Fernanda Esposito, Herrison Fontana, Bruna Fuga, Adriana Cardenas-Arias, Quézia Moura, Brenda Cardoso, Gladyston C.V. Costa, Tatiana C.M. Bosqueiro, Juliana A. Sinhorini, Eduardo de Masi, Caroline C. Aires, Nilton Lincopan

**Affiliations:** a Department of Microbiology, Institute of Biomedical Sciences, University of São Paulo, São Paulo, Brazil; b Zoonoses Surveillance Division, Health Surveillance Coordination, Municipal Health Department, São Paulo, Brazil; c One Health Brazilian Resistance Project (OneBR), Brazil; d Department of Clinical Analysis, School of Pharmacy, University of São Paulo, São Paulo, Brazil; e Federal Institute of Espírito Santo, Vila Velha, Brazil

**Keywords:** Enterobacterales, Antimicrobial resistance, ESBL, Resistome, Urban wildlife, Rats, Pigeons, Genomic surveillance, One health

## Abstract

WHO priority pathogens have disseminated beyond hospital settings and are now being detected in urban and wild animals worldwide. In this regard, synanthropic animals such as urban pigeons (*Columba livia*) and rodents (*Rattus rattus*, *Rattus norvegicus* and *Mus musculus*) are of interest to public health due to their role as reservoirs of pathogens that can cause severe diseases. These animals usually live in highly contaminated environments and have frequent interactions with humans, domestic animals, and food chain, becoming sentinels of anthropogenic activities. In this study, we report genomic data of *Escherichia coli* strains selected for ceftriaxone and ciprofloxacin resistance, isolated from pigeons and black rats. Genomic analysis revealed the occurrence of international clones belonging to ST10, ST155, ST224 and ST457, carrying a broad resistome to beta-lactams, aminoglycosides, trimethoprim/sulfamethoxazole, fluoroquinolones, tetracyclines and/or phenicols. SNP-based phylogenomic investigation confirmed clonal relatedness with high-risk lineages circulating at the human-animal-environmental interface globally. Our results confirm the dissemination of WHO priority CTX-M-positive *E. coli* in urban rodents and pigeons in Brazil, highlighting potential of these animals as infection sources and hotspot for dissemination of clinically relevant pathogens and their resistance genes, which is a critical issue within a One Health perspective.

## Highlights

• Synanthropic pigeons and rodents as source of AMR bacteria were investigated.
• Identification of One Health global clones of CTX-M-positive *E.coli* is highlighted.
• Phylogenomic analysis revealed clonal relatedness with hospital-associated lineages.
• Phylogeographical analysis confirmed expansion of ST392 as global One Health clone.
• Synanthropic animals could be indicators of AMR burden in anthropized environments.

## Graphical abstract

**Figure:**
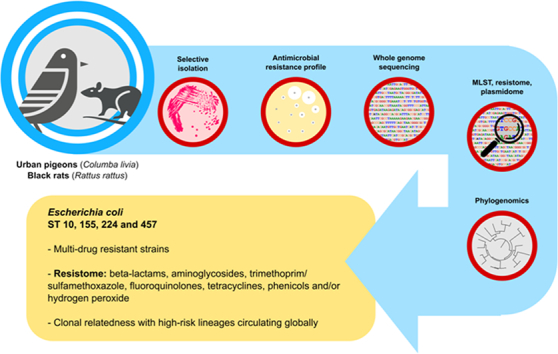

## 1 Introduction

WHO critical priority pathogens, which include ESBL-producing Enterobacterales, have broken the boundaries of hospital settings and their prevalence in both hospital- and community-acquired infections is on a rise [1,2,3]. Moreover, ESBL-producing *Escherichia coli* has been lately reported in wild animals, including urban wildlife [1,4,5,6]. In this regard, CTX-M positive *E. coli* has become a threat to global health and a One Health challenge, due to its resistance to several commonly used antibiotics, its ability to colonize or infect different hosts either through contact or through food/water contamination, presence in different environments, rapid spread, and worldwide distribution [1]. Synanthropic animals, such as urban pigeons (*Columba livia*) and rodents (*Rattus norvegicus*, *Rattus rattus* and *Mus musculus*) are of interest in public health due to their frequent interactions with humans, animals and food chains, their habits of foraging in human leavings and garbage and sheltering in highly contaminated environments, such as sewers and polluted river banks (in the case of rats), and their role as reservoirs of zoonotic agents that can cause severe human and animal disease [7,8,9]. Such characteristics also make synanthropic animals sentinels of anthropogenic activities, being potential indicators of clinically relevant drug-resistant Enterobacterales, such as CTX-positive *E. coli*, beyond hospital settings ([10]; [[11], [12], [13], [14], [15], [16]]; [17]; [18]). In this study, we report genomic data and phylogenomic analysis of multidrug-resistant *E. coli*, including four CTX-M-positive isolates from feral pigeons and black rats.

## 2 Materials and methods

### 2.1 Field capture and sample collection

Between September 2018 and November 2019, biologists of the Zoonoses Surveillance Division of São Paulo city, Brazil, captured 22 feral urban rodents (1 *R. norvegicus* and 21 *R. rattus*), and 22 diseased (*n* = 15) or dead (*n* = 7) feral urban pigeons (*C. livia*) within the Division's headquarters and surrounding areas. Rodents were captured using cage traps in rodent control activities. By the time of collection, 16 rodents were living and 6 were dead. Rectal and oral samples were collected, as well as feces deposited in the trap (when available). Species identification of the rats were made by rodent specialist's biologists working in the Nucleus of Surveillance and Control of Synantropic Animals at the Center for Zoonoses Control, in São Paulo; using guidelines provided by the Brazilian Ministry of Health (https://bvsms.saude.gov.br/bvs/publicacoes/manual_roedores1.pdf). In this regard, *R. norvegicus* and *R. rattus* were captured in an urban area (Table 1) near to drainage canals and the Tietê River, which is affected by the direct discharge of anthropogenic pollutants (including domestic sewage and hospital wastewater) in São Paulo, the largest and most populous city in Brazil. Pigeons were collected during the investigation of a Newcastle Disease Virus (NDV) outbreak [19]. Cloacal and ingluvial samples were collected from 15 diseased and 7 dead pigeons, using transport swab with Amies and charcoal medium (Copan Italia, Brescia, Italy).

**Table 1:** Microbiological and genomic characteristics of *E. coli* strains isolated from rats and pigeons in São Paulo, Brazil, 2019.

Strain	P2C1	P22C2	P32P1	R6R1	R7F1
Host	Pigeon (*Columba livia*)	Pigeon (*Columba livia)*	Pigeon (*Columba livia*)	Black rat (*Rattus rattus*)	Black rat (*Rattus rattus*)
Sample	Cloacal swab	Cloacal swab	Cloacal swab	Rectal swab	Feces
Sampling location	23.512548°S	23.512308°S	23.511926°S	23.512308°S,	23.512273°S
	46.628033°W	46.627528°W	46.627990°W	46.627528°W	46.627326°W
Antibiotic resistance profile(MIC)^a^	GEN, ATM, SUT, CFO, CPM, CAZ, CRO, CTX, AMC	SUT, CIP (4), ENO (4), LVX (2), CPM, CRO, TET	GEN, ATM, SUT, CIP (16), ENO (16), LVX (4), CPM, CRO, CTX, TET	ATM, SUT, CPM, CRO, TET	SUT, CIP (4), ENO (4), LVX (2), TET
Genome size	5,057,165 bp	4,816,687	4,928,895	4,854,079	4,717,418
No. Of CDS^b^	4684	4513	4556	4539	4361
G + C content	50.45%	50.62%	50.96%	50.63%	50.78%
rRNAs/tRNAs/tmRNAs	3/57/1	4/50/1	3/82/1	4/80/1	3/50/1
MLST^c^	ST457	ST10	ST224	ST155	ST10
*fimH* type	145	30	61	121	54
Serotype	O11:H6	O101:H10	O126:H23	O32:H21	O107:H10
Phylogroup	F	A	B1	B1	A
Resistome					
Aminoglycosides	*aph(3″)-Ib*, *aph(6)-Id*	*aph(3″)-Ib*, *aph(6)-Id*	*aac(3)-IId, aph(3″)-Ib*, *aph(3′)-Ia, aph(6)-Id*	*aadA5*	*aadA2*
Beta-lactams	*bla*_CTX-M-2_,*bla*_CMY-2_, *bla*_TEM-1B_	*bla*_CTX-M-8_	*bla*_CTX-M-1_	*bla*_CTX-M-8_	*bla*_TEM- 1B_
Fluoroquinolones	*gyrA*(S83L)	*gyrA* (S87L, D87N), *parC* (S80I)	*gyrA* (S87L, D87N), *parE* (S458A)	–	*qnrB19*, *gyrA* (S83L), *parC* (S80I)
Sulfonamides	*sul2*	*sul1, sul2*	*sul1, sul2*	*sul2*	*sul3*
Trimethoprim	–	*dfrA1*	*dfrA7*	*dfrA17*	*dfrA12*
Tetracyclines	–	*tetA*	*tetA*	*tetA*	*tetA*
Phenicols	–	*floR*	–	*cmlA1*	–
Macrolides	*mphB*	*mphB*	*mphA*	–	*–*
Hydrogen peroxide tolerance	*sitABCD*	*sitABCD*	–		–
Plasmidome	IncFII, IncFIB, ColRNAI	IncFIA, IncFIB, IncFIC, IncI1-I, IncQ1, Col(MG828)	IncQ1, p0111	IncI1-I, IncI2, IncY	IncX1, Col(MG828), Col(pHAS28), Col440I, ColRNAI
GenBank accession number	JAAVLE000000000.1	JAKVRA000000000.1	JAKVDG000000000.1	JAAVLD000000000.1	JAKVDH000000000.1
ONEBr ID	ONE49	ONE130	ONE131	ONE01	ONE129

a MIC, minimal inhibitory concentration (μg/mL); GEN, gentamycin; AMC, amoxicillin/clavulanate; ATM, aztreonam; CAZ, ceftazidime; CFO, cefoxitin; CIP, ciprofloxacin; CPM, cefepime; CRO, ceftriaxone; CTX, cefotaxime; ENO, enrofloxacin; GEN, gentamycin; LVX, levofloxacin; SUT, cotrimoxazole, TET, tetracycline.

b CDS, coding sequence.

c MLST, multi-locus sequence type; ST, sequence type.

### 2.2 Bacterial culture, identification and antibiogram

Swabs were inoculated on MacConkey agar and incubated at 35 ± 1 °C for 24 h, and lactose-fermenting colonies were identified by MALDI-TOF (Bruker MALDI Biotyper CA System) [20]. *E. coli* isolates were then inoculated on two MacConkey agar plates, one supplemented with ceftriaxone (2 μg/mL) and the other with ciprofloxacin (2 μg/mL). Plates were incubated at 35 ± 1 °C for 24 h.Antimicrobial susceptibility tests were performed by disc diffusion method, according to guidelines [21]. The following antibiotics were tested: amikacin, amoxicillin/clavulanate, aztreonam, cefepime, cefotaxime, cefoxitin, ceftazidime, ceftriaxone, ciprofloxacin, cotrimoxazole, ertapenem, fosfomycin, gentamycin, imipenem, meropenem and tetracycline. Minimum inhibitory concentrations (MICs) for three fluoroquinolones (ciprofloxacin, enrofloxacin and levofloxacin) were determined for strains that presented ciprofloxacin resistance by the disc diffusion method. Interpretation of both disc diffusion and MIC results were made according to CLSI guidelines [22,21]. Extended-spectrum beta-lactamases (ESBL) production was observed through double disk synergy test, using amoxicillin/clavulanate, ceftriaxone, ceftazidime, cefotaxime and cefepime.

### 2.3 Whole genome sequencing

Genomic DNAs from all ESBL-producing *E. coli* strains, isolated from pigeons (P2C1, P22C2 and P32P1) and rodent (R6R1), and from the ciprofloxacin-resistant *E. coli* strain R7F1 isolated from rodent were extracted using PureLink Quick Gel Extraction & PCR Purification Combo Kit (Life Technologies, Carlsbad, CA, USA). The Illumina paired-end libraries were constructed using a Nextera XT DNA Library Preparation Kit (Illumina Inc., Cambridge, UK), according to the manufacturer's guidelines. Whole genome sequencing (WGS) was then performed using Illumina MiSeq or NextSeq platform. Paired reads had adaptors trimmed using Trim Galore 0.6.6 (https://github.com/FelixKrueger/TrimGalore), then the trimmed reads were assembled into contigs using Unicycler 0.4.8 (https://github.com/rrwick/Unicycler). Genome assemblies were annotated using Prokka v1.14.6 (https://github.com/tseemann/prokka). Center for Genomic Epidemiology tools (http://www.genomicepidemiology.org) were used to confirm species (SpeciesFinder v2.0) and to identify multi-locus sequence type (MLST v2.0), resistance genes (Resfinder v4.1), plasmid replicons (PlasmidFinder v2.1), *fimH* type (FimTyper v1.0), and O-antigen and H-flagellar antigen types (SerotypeFinder v2.0). Clermont phylogroups were identified using EZClermont v0.7.0 (https://github.com/nickp60/ezclermont). ESBL genetic context and plasmids annotations were manually curated with Geneious Prime R10 software using the Uniprot (https://www.uniprot.org/blast) and ISFinder (https://isfinder.biotoul.fr) databases, whereas comparative analysis were performed with nucleotide BLAST alignment. Schematic presentation of the genetic environment and plasmids were performed using EasyFig v.2.2.2 (https://mjsull.github.io/Easyfig).

### 2.4 Phylogenomic analysis

In order to compare the strains isolated from rats and pigeons with phylogenetically related strains, a search for sequence types (ST) ST10, ST155, ST224 and ST457 was performed on the *Shigella*/*Escherichia* database in Enterobase (https://enterobase.warwick.ac.uk). Genome assemblies and metadata of isolates with the same ST and *fimH* type of each strain were downloaded from Enterobase, ignoring isolates with no data for country, source of isolation and/or collection year. FastANI v1.32 (https://github.com/ParBLiSS/FastANI) was used to assess average nucleotide identity (ANI) between the 5 strains and the corresponding downloaded genomes from Enterobase. The 30 genomes with highest ANI with each of the 5 strains were selected for phylogenomic analysis. In this regard, we used default settings of CSI Phylogeny v1.4 (https://cge.cbs.dtu.dk/services/CSIPhylogeny) to generate four maximum-likelihood phylogenetic trees (one for each ST). Chromosome sequences of *E. coli* ST10 strain LD27–1 (RefSeq accession number: NZ_CP047594.1), ST155 strain WAT (NZ_CP012380.1), ST224 strain MS14386 (NZ_LR130552.1) and ST457 strain CREC-544 (NZ_CP024826.1) were used as reference for each analysis. ABRicate v1.0.1(https://github.com/tseemann/abricate) was used with ResFinder (https://bitbucket.org/genomicepidemiology/resfinder_db) and PlasmidFinder (https://bitbucket.org/genomicepidemiology/plasmidfinder_db) databases to screen all genomes within phylogenomic analysis for resistance genes and plasmid replicons, respectively. Mutations on quinolone resistance-determining regions (QRDR) were identified using PointFinder v3.1.1 (https://bitbucket.org/genomicepidemiology/pointfinder). Each tree was rooted at midpoint using iTOL v6 (https://itol.embl.de), which was also used to annotate the trees with data from Enterobase (Table S1) and ABRicate.

## 3 Results and discussion

**Fig. 1:**
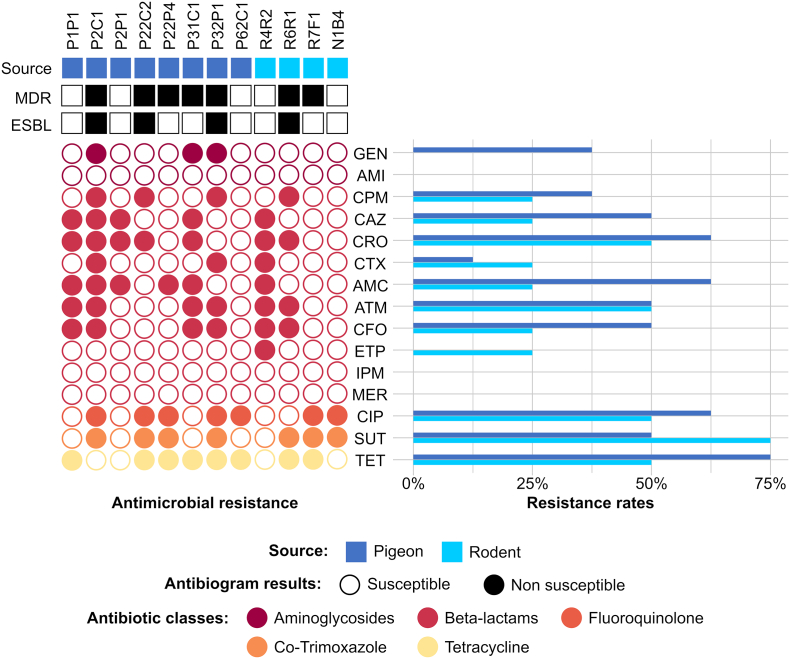
Results of antimicrobial susceptibility testing for each *Escherichia coli* isolate from pigeons and rodents obtained in ceftriaxone- and ciprofloxacin-supplemented agar, as well as resistance rates for each tested antibiotic.

Four *E. coli* strains from 4 different rodents were isolated using drug-supplemented agar. In this regard, while 2 *E. coli* were recovered from ceftriaxone-supplemented agar, another 2 isolates were recovered from ciprofloxacin-supplemented agar. From pigeon samples, 8 *E. coli* strains were isolated from 6 different birds, of which 3 isolates have grown on both drug-supplemented plates. Additionally, 3 *E. coli* isolates have grown only on ceftriaxone-containing agar plates, and other 2 isolates were isolated from ciprofloxacin-containing agar plates. Antimicrobial susceptibility tests were performed for all 12 isolates (Fig. 1).

Higher rates of antimicrobial resistance were observed in pigeon isolates than in rodent isolates (Fig. 1). Moreover, ESBL-producing isolates were observed in 9.1% of rodents and 13.1% of pigeons. Higher resistance rates and prevalence in pigeons is possibly due to the fact that pigeons collected for this study were captured in an NDV outbreak context, and all of them were diseased or dead, thus probably more susceptible to opportunist pathogens.

Isolates displaying resistance to 3 or more classes of antibiotics were considered multi-drug resistant (MDR) [23]. For WGS analysis 5 *E. coli* isolates were selected based on a MDR profile for broad-spectrum cephalosporins, ciprofloxacin, co-trimoxazole (trimethoprim–sulfamethoxazole), tetracycline or gentamicin (Fig. 1; Table 1): R6R1, a rectal isolate from a black rat captured dead; and R7F1, isolated from rat feces; P2C1, a cloacal isolate from a diseased NDV-positive pigeon; P22C2, a cloacal isolate from a non-tested diseased pigeon; and P32P1, an ingluvial isolate from a diseased NDV-negative pigeon.

In this study, the occurrence of ST10, ST155, ST224 and ST457 was confirmed in urban rats and pigeons (Table 1). In this respect, ST10 is a global *E. coli* lineage, found in different hosts, associated with extraintestinal pathogenicity that may carry different types of plasmids containing resistance genes [24,25]. In Brazil, high-risk clones of *E. coli* belonging to ST10 has been mostly identified in environmental and animal sources, including coastal waters, penguins, and cattle [26,27]. ST155 has been recently described as a potential food-borne pathogen, since it can cause disease in both poultry and humans [28,29,30]; having been recently identified in Brazil in humans, urban-impacted coastal waters and river fishes [31,26,32].

ST224 is a global clone frequently described in food production animals and retail food [33,34], as well as wild animals [35,36], having also been reported causing urinary tract infection in humans [37]. In Brazil, ST224 was identified in swine, companion animals and river fishes [32,34,38]. The ST457 has been recently described as an emerging extraintestinal pathogenic *E. coli* found mainly in wildlife and in food-producing animals [39], having also been reported in marine environment in Brazil [40], whereas transmission from companion animals to humans has been documented in China [41]. Moreover, this lineage has been associated to bloodstream infections [42].

ESBL-producing Enterobacterales has an endemic status in Brazil [43], where *bla*_CTX-M-2_, *bla*_CTX-M-8_ and *bla*_CTX-M-15_ are the most prevalent variants identified in healthcare- and community-associated Enterobacterales infections ([[44], [45]]). Moreover, non-human hosts such as cattle [27], poultry and chicken meat [[46], [47], [48]] and synanthropic birds [12] has also been described as sources of CTX-M-producing *E. coli* in different Brazilian cities.

**Fig. 2:**
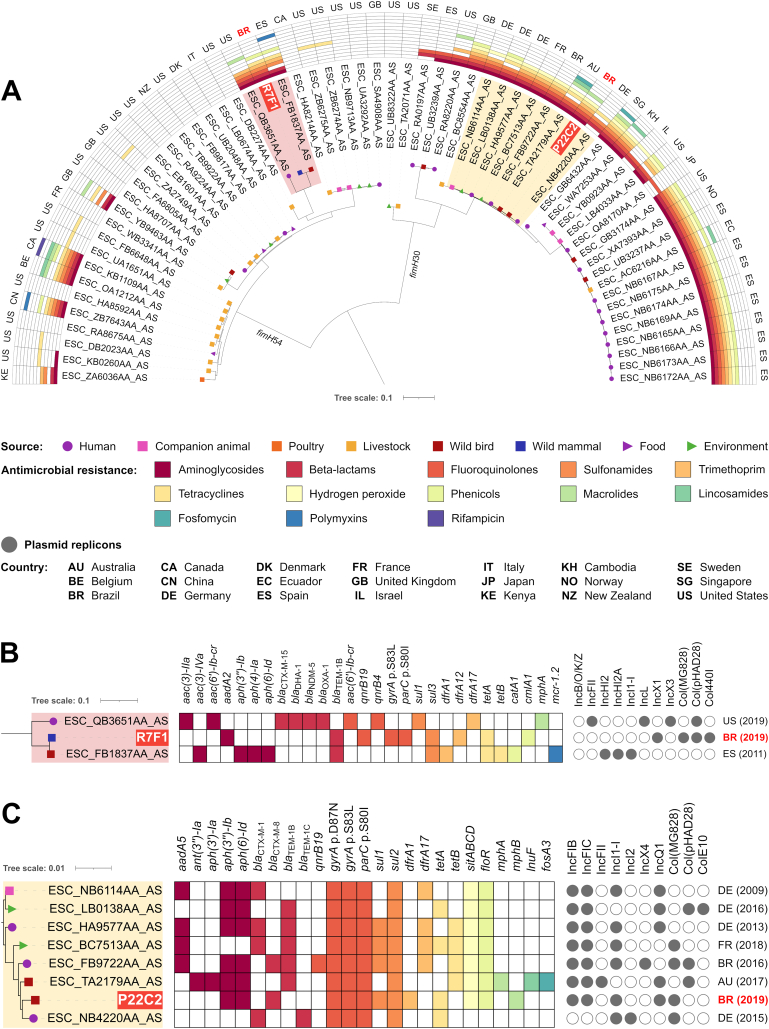
In A, phylogenetic tree plotted in a 180° arc, with 62 *Escherichia coli* ST10 strains, their isolation source, presence/absence of antimicrobial resistance genes for different antimicrobial classes, and country of collection. In B, highlighted cluster in “A” with *fimH*54 strains, their source of isolation, resistome, plasmidome, country and year of collection. In C, highlighted cluster in “A” with *fimH*30 strains, their source of isolation, resistome, plasmidome, country and year of collection.

On phylogenomic analysis, ST10 strains formed two clades, one for each *fimH* type, 30 and 54 (Fig. 2). Percentage of reference genome covered by all isolates was 76.97%, corresponding to 3,612,870 positions found in all analyzed genomes, and SNP counts ranged from 0 to 10,152 (Table S2).

On *fimH*54 clade, which includes R7F1 strain, SNP differences ranged from 0 to 3698, and half of the isolates had no resistance gene identified (Fig. 2). R7F1 strain is genetically close (186 SNPs) to a MCR-1-producing wild bird isolate from Spain, but resistomes and plasmidomes are different, *e.g.*, while the isolate from Spain has no resistance gene for fluoroquinolones, R7F1 has the *qnrB19* gene and mutations in QRDR, which highlights the importance of both horizontal transfer of resistance genes in mobile genetic elements and vertical transfer of mutations in resistance-determining regions for antimicrobial resistance spreading. On the other hand, on ST10*fimH*30 clade, which includes P22C2 strain, SNP counts ranged between 1 and 3678 (Table S2). Most of the isolates within *fimH*30 clade presented resistance genes for aminoglycosides, beta-lactams, fluoroquinolones, sulfonamides, trimethoprim, tetracyclines and/or hydrogen peroxide, as well as mutations in QRDR. P22C2 grouped with strains from human, companion animal, wild bird and environmental sources, mostly CTX-M-1 or CTX-M-8 producers, from Germany, France, Brazil, and Australia, with 57 and 185 SNP differences (Table S2).

**Fig. 3:**
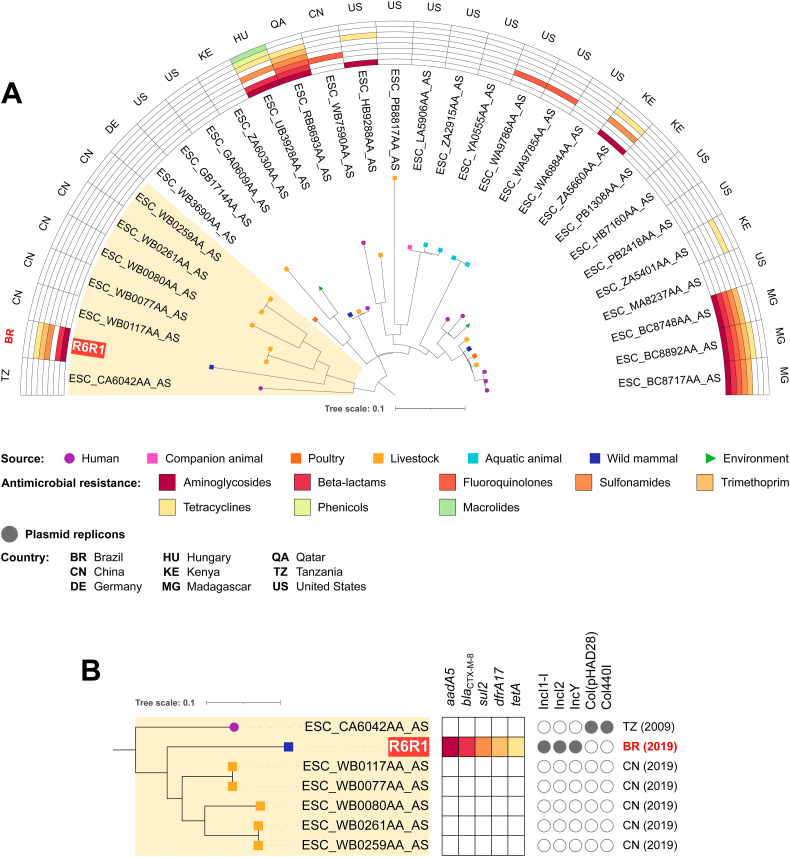
In A, phylogenetic tree plotted in a 180° arc, with 31 *Escherichia coli* ST155 *fimH*121strains, their isolation source, presence/absence of antimicrobial resistance genes for different antimicrobial classes, and country of collection. In B, highlighted cluster in “A”, with source of isolation, resistome, plasmidome, country and year of collection for each strain.

Phylogenomic analysis of ST155 *fimH*121 revealed high diversity among strains, with 0 to 3047 SNP differences (Table S3). Percentage of reference genome covered by all genomes was 87.78%, corresponding to 4,254,315 positions found in all genomes. R6R1 grouped with relatively distant strains: 5 livestock isolates from China, next a human isolate from Tanzania. SNP count among strains within this clade ranged from 0 to 2247 (Table S3). Strikingly, while R6R1 presents resistance genes for aminoglycosides, beta-lactams, sulfonamides, trimethoprim and tetracyclines, we did not identify any resistance gene in the other strains within the clade (Fig. 3). The presence of Inc-type plasmids was only observed in R6R1, highlighting the importance of horizontal transfer.

**Fig. 4:**
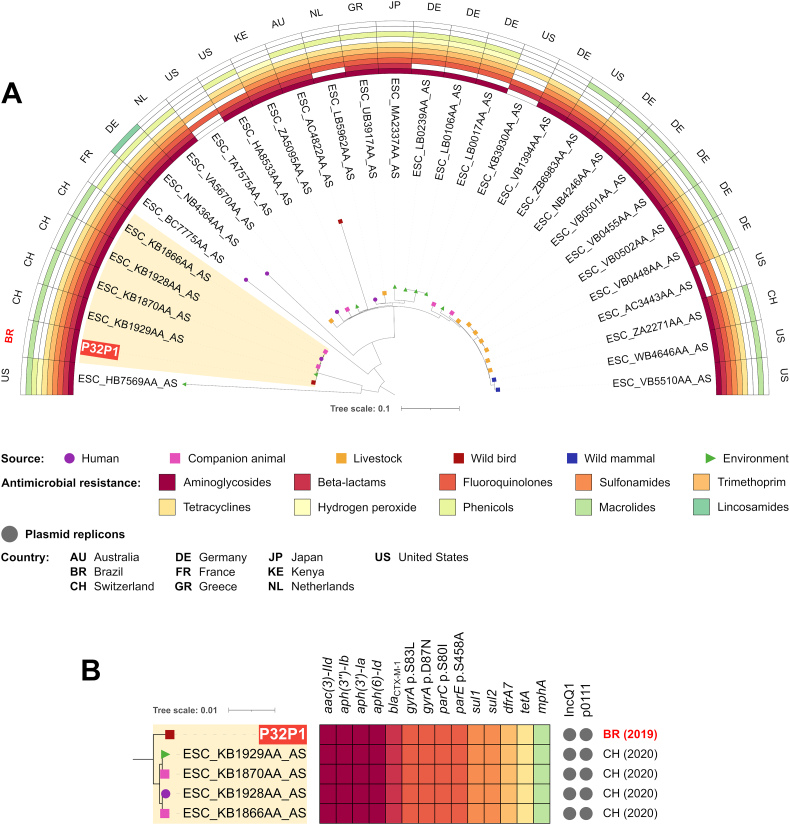
In A, phylogenetic tree plotted in a 180° arc, with 31 *Escherichia coli* ST224 *fimH*61 strains, their isolation source, presence/absence of antimicrobial resistance genes for different antimicrobial classes, and country of collection. In B, highlighted cluster in “A”, with source of isolation, resistome, plasmidome, country and year of collection for each strain.

Within ST224 *fimH*61 strains, 90.99% of reference genome was covered by all isolates, corresponding to 4,405,087 positions. SNP differences ranged between 0 and 1288 (Table S4). Phylogenomic analysis revealed that P32P1 is genetically very close to 4 strains from Switzerland, isolated from companion animal, human and environmental sources, sharing the same resistome and plasmidome (Fig. 4). Within this clade, SNP counts ranged from 3 to 16 (Table S4), which suggest this lineage is able to clonally spread to different hosts, highlighting a zoonotic/zooanthroponotic potential and the possible role of pigeons as reservoirs.

**Fig. 5:**
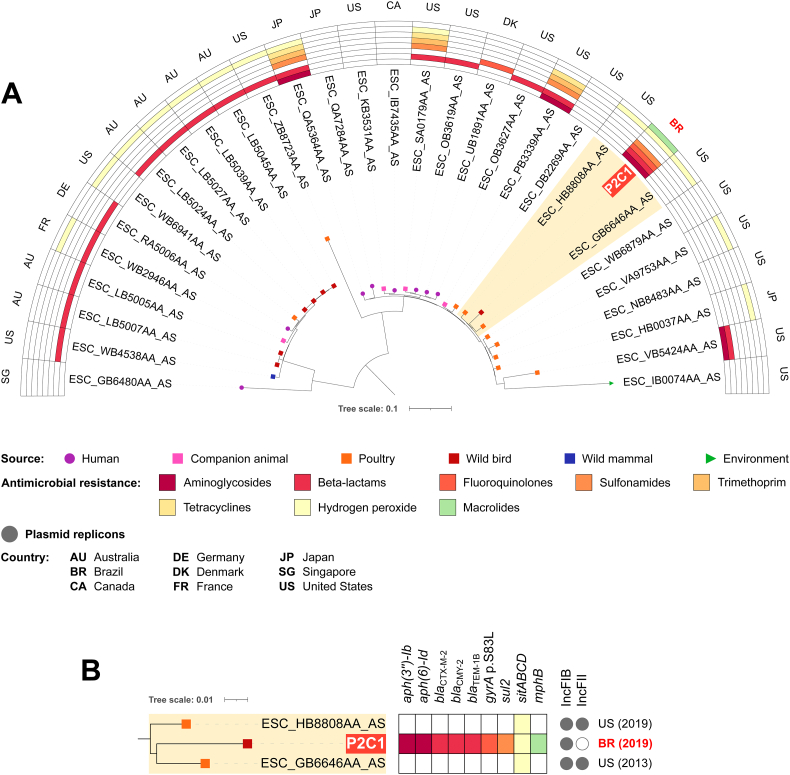
In A, phylogenetic tree plotted in a 180° arc, with 31 *E. coli* ST457 *fimH*145 strains, their isolation source, presence/absence of antimicrobial resistance genes for different antimicrobial classes, and country of collection. In B, highlighted cluster in “A”, with source of isolation, resistome, plasmidome, country and year of collection for each strain.

Finally, on phylogenomic analysis of ST457 *fimH*145, 91.46% of the reference genome was covered by all isolates, corresponding to 4,480,690 positions. SNP differences ranged between 1 and 2427 (Table S5). P2C1 grouped with 2 poultry isolates from United States, with SNP counts of 250 and 265 (Table S5). While P2C1 presents resistance genes for aminoglycosides, beta-lactams, sulfonamides, in the 2 closest isolates we only identified the *sitABCD* locus, related to hydrogen peroxide tolerance (Fig. 5).

**Fig. 6:**
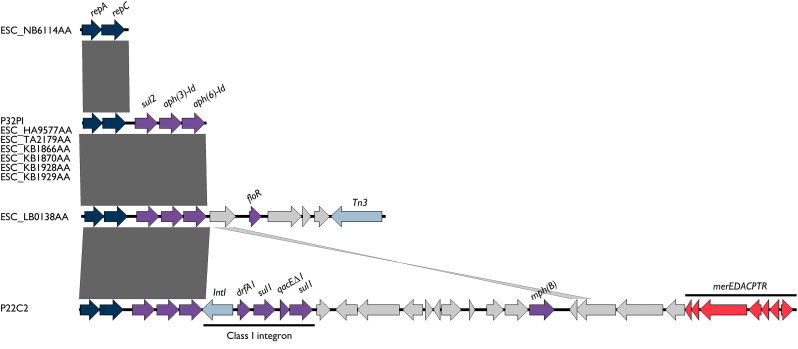
Schematic representation of IncQ1 plasmids identified in *E. coli* ST10 and ST224. Arrows represents coding sequences and are labelled by color as follows: purple, antimicrobial resistance genes; red, disinfectants compounds or heavy metal resistance; shadow blue, plasmid replication proteins; light blue, mobile genetic elements; gray, hypothetical proteins or other genes. Dark gray shadows represent regions of homology. (For interpretation of the references to color in this figure legend, the reader is referred to the web version of this article.)

Plasmid analysis of phylogenetically related *E. coli* ST10 and ST254 showed that IncQ1 plasmids are widespread in those strains, and usually carries aminoglycosides and sulfonamides resistance genes. However, an IncQ1 plasmid from ESC_LB0138AA additionally carried a chloramphenicol/florfenicol efflux MFS transporter *floR* gene and thus regarded as multidrug-resistance (MDR) plasmid. On the other hand, our pigeon strain P22C2 harbored a MDR IncQ1 plasmid carrying macrolide 2′-phosphotransferase II [*mph(B)*], a class I integron containing *drfA1*-*sul1*-*qacEΔ1*-*sul2* genetic array, and the mercury resistance operon (*merEDACPTR*) (Fig. 6).

**Supplementary Fig. S1:**
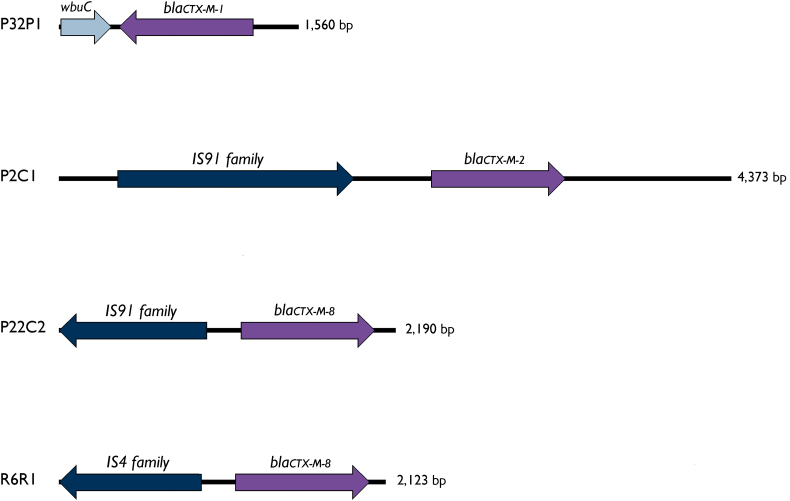
Genetic context of *bla*_CTX-M_ genes in *E. coli* ST10, ST155, ST224 and ST457 strains isolated from urban rodents and pigeons.

Due to limitations of short-read sequencing technology, it was not possible to obtain complete nucleotide sequences of the plasmids, but further analysis revealed that IncQ1 plasmids were found to carry antimicrobial, disinfectants, and heavy metal resistance genes in P22C2 and P32P1(Fig. 6). The ESBL CTX-M-types identified in this study were in contigs of short length, and thus its precise location could not be determined. However, in P22C2 and P2C1, *bla*_CTX-M-2_ and *bla*_CTX-M-8_ genes were mediated by IS*91* family transposase, whereas in R6R1, *bla*_CTX-M-8_ was identified upstream of a IS*4* insertion sequence. The *bla*_CTX-M-1_ gene was located 46 bp downstream of a cupin fold metalloprotein (*wbuC*) in P32P1 strain (Fig. S1).

Furthermore, in R7F1, *bla*_TEM-1B_ gene was localized in a large contig (86.704 bp) and was closely related (99.93% nucleotide identity; 90% query coverage) to a complete chromosome of a *E. coli* ST10 of bovine origin from Switzerland (Genbank accession number: CP038791.1), thus suggesting its integration in the chromosome. On the other hand, in P2C1, the *bla*_TEM-1B_ gene contig (10,012 bp) was highly similar (99.32% nucleotide identity and 99% query coverage) to IncFII p4_4.2 plasmid (Genbank accession number: CP023828.1) in *E. coli* from human*.*

P2C1 also harbored the AmpC CMY-2 gene identified within IS*91*-*orf*-*bla*_CMY-2_-*blc*-*sugE* genetic environment, along with aminoglycoside resistance genes [*aph(3″)-Ib*, *aph(6)-Id*] in a contig of 7.583 bp in length displaying 100% nucleotide identity and 88% query coverage to an CMY-positive plasmid from *Salmonella enterica* ser. Anatum (Genbank accession number: CP045467.1).

Although this study has limitations such as convenience sampling and a small number of animals collected in just one place, the detection of ESBL-producing Enterobacterales in such animals is of concern, due to the proximity of synanthropic animals and human populations in urban environment. However, with this dataset it is not possible to neither compare different regions, nor analyze correlations with socioeconomic features. Therefore, studies with probabilistic sampling are needed in order to assess the role urban wildlife play in antimicrobial resistance spreading and transmission. Animals and carcasses collected in pest control activities may be valuable to elucidate how these animals may acquire, carry, spread and transmit antimicrobial resistant microorganisms into their life range.

In summary, synanthropic animals such as urban rats and pigeons are frequently exposed to contaminated places they use for shelter or foraging. Their behavior favors frequent interactions with humans, animals (companion, food-producing and wild), food chains, urban waterways, sewage, and domestic garbage, making them potential reservoirs and transmitters of antimicrobial resistant pathogens. Despite limitations in this study, phylogenomic results corroborates this hypothesis, once the isolates from rats and pigeons are closely related to isolates from different hosts and sources, such as humans, companion animals, environments, livestock, poultry, and other wild animals, suggesting that these animals may contribute with dissemination of WHO priority pathogens through the human-animal-environment interface, which is a critical issue within a One Health perspective.

The following are the supplementary data related to this article.

Supplementary Table S1 Metadata for each Enterobase downloaded genome used on phylogenomic analyses. Supplementary Table S1

Supplementary Table S2 SNP counts for *Escherichia coli* ST10 isolates. Supplementary Table S2

Supplementary Table S3 SNP counts for *Escherichia coli* ST155 isolates. Supplementary Table S3

Supplementary Table S4 SNP counts for *Escherichia coli* ST457 isolates. Supplementary Table S4

Supplementary Table S5 SNP counts for *Escherichia coli* ST224 isolates. Supplementary Table S5

## Accession numbers

This Whole Genome Shotgun project has been deposited at DDBJ/ENA/GenBank under the accession numbers JAAVLE000000000.1 (P2C1), JAKVRA000000000.1 (P22C2), JAKVDG000000000.1 (P32P1), JAAVLD000000000.1 (R6R1) and JAKVDH000000000.1 (R7F1). Genomic data has been deposited at OneBR (http://onehealthbr.com), under IDs ONE01 (R6R1), ONE49 (P2C1), ONE129 (R7F1), ONE130 (P22C2) and ONE131 (P32P1).

## Author contributions

NL and ES contributed to conception and design of this study. ES, GC, TB, JS and EM participated in animal capture. ES, JS, LO, HM and CA contributed to sample collections. ES, FE and BC performed isolation, antibiogram, screening and maintenance procedures. FAO and CL identified the isolates species by Matrix Associated Laser Desorption-Ionization - Time of Flight, AC and QM performed DNA extraction, DNA quality and DNA preparation for WGS. FE, BF and ES assembled the genomes from the raw reads and made *in silico* analyses. ES made the phylogenomic analysis. HF made the plasmid and genetic environment analyses. All authors contributed to manuscript revision, read, and approved the submitted version.

## Funding

This work was supported, in whole or in part, by the 10.13039/100000865Bill & Melinda Gates Foundation [Grand Challenges Explorations Brazil OPP1193112]. Under the grant conditions of the Foundation, a CC BY or equivalent licence is applied to the Author Accepted Manuscript version arising from this submission. Additionally, this study was supported by the 10.13039/501100001807Fundação de Amparo à Pesquisa do Estado de São Paulo (2020/08224-9) and 10.13039/501100003593Conselho Nacional de Desenvolvimento Científico e Tecnológico (AMR 443819/2018-1, 433128/2018-6 and 422984/2021-3). B.C. is a research fellow of 10.13039/501100002322CAPES (88882.333054/2019-01), F.E. is a research fellow of 10.13039/501100001807FAPESP (2019/15578-4), and B.F. is a research fellow of 10.13039/501100002322Coordenação de Aperfeiçoamento de Pessoal de Nível Superior (CAPES, 88887.358057/2019-00). N.L. is a research fellow of 10.13039/501100003593CNPq (314336/2021-4).

## Author statement

We declare that the manuscript “*One Health clones of multidrug-resistant Escherichia coli carried by synanthropic animals in Brazil*” by Elder Sano, Fernanda Esposito, Herrison Fontana, Bruna Fuga, Adriana Cardenas-Arias, Quézia Moura, Brenda Cardoso, Gladyston C. V. Costa, Tatiana C. M. Bosqueiro, Juliana A. Sinhorini, Eduardo de Masi, Caroline C. Aires, and Nilton Lincopan, has not been published before and is not under consideration for publication elsewhere.

All authors made relevant contributions to the development of the research, the manuscript has been read and approved by all named authors and confirm that the order of authors listed in the manuscript has been approved by all of us. We also affirm that there are no known conflicts of interest associated with this publication and there has been no significant financial support for this work that could have influenced its outcome.

We understand that the Corresponding Author is the sole contact for the Editorial process. He is responsible for communicating with the other authors about progress, submissions of revisions and final approval of proofs. We confirm that we have provided a current, correct email address.

We are providing details in the table below of each author contribution to the submitted manuscript:

**Table:** 

Authors	Conception and design of study	Acquisition of data: laboratory or clinical	Analysis of data	Drafting of article and/or critical revision	Final approval of manuscript
Elder Sano	X	X	X	X	X
Fernanda Esposito	X	X	X	X	X
Herrison Fontana		X	X	X	X
Bruna Fuga			X	X	X
Adriana Cardenas-Arias		X		X	X
Quézia Moura		X	X	X	X
Brenda Cardoso		X		X	X
Gladyston C. V. Costa		X	X	X	X
Tatiana C. M. Bosqueiro		X		X	X
Juliana A. Sinhorini		X		X	X
Eduardo de Masi		X		X	X
Caroline C. Aires		X		X	X
Nilton Lincopan	X	X	X	X	X

## Declaration of Competing Interest

The authors declare that the research was conducted in the absence of any commercial or financial relationships that could be construed as a potential conflict of interest.

## Data Availability

Genomic data ae publicly available on NCBI

## References

[1] Bezabih Y.M., Sabiiti W., Alamneh E., Bezabih A., Peterson G.M., Bezabhe W.M. (2021). The global prevalence and trend of human intestinal carriage of ESBL-producing *Escherichia coli* in the community. J. Antimicrob. Chemother..

[2] Kawamura K., Nagano N., Suzuki M., Wachino J., Kimura K., Arakawa Y. (2017). ESBL-producing *Escherichia coli* and its rapid rise among healthy people. Food Safety.

[3] Woerther P.L., Burdet C., Chachaty E., Andremont A. (2013). Trends in human fecal carriage of extended-spectrum β-lactamases in the community: toward the globalization of CTX-M. Clin. Microbiol. Rev..

[4] Hassell J.M., Ward M.J., Muloi D., Bettridge J.M., Robinson T.P., Kariuki S. (2019). Clinically relevant antimicrobial resistance at the wildlife–livestock–human interface in Nairobi: an epidemiological study. Lancet Planet. Health.

[5] Leu-Burke G., Miller T., Fowler S. (2019).

[6] Palmeira J.D., Cunha M.V., Carvalho J., Ferreira H., Fonseca C., Torres R.T. (2021). Emergence and spread of cephalosporinases in wildlife: a review. Animals.

[7] Himsworth C.G., Parsons K.L., Jardine C., Patrick D.M. (2013). Rats, cities, people, and pathogens: a systematic review and narrative synthesis of literature regarding the ecology of rat-associated zoonoses in urban centers. Vector-Borne Zoonot. Diseas..

[8] Morand S., Jittapalapong S., Kosoy M. (2015). Rodents as hosts of infectious diseases: biological and ecological characteristics. Vector Borne Zoonot. Diseas..

[9] Torres-Mejía A.M., Blanco-Peña K., Rodríguez C., Duarte F., Jiménez-Soto M., Esperón F. (2018). Zoonotic agents in feral pigeons (*Columba livia*) from Costa Rica: possible improvements to diminish contagion risks. Vector-Borne Zoonot. Diseas..

[10] Ben Yahia H., Ben Sallem R., Tayh G., Klibi N., Ben Amor I., Gharsa H., Boudabbous A., Ben Slama K. (2018). Detection of CTX-M-15 harboring *Escherichia coli* isolated from wild birds in Tunisia. BMC Microbiol..

[11] Borges C.A., Maluta R.P., Beraldo LG, Cardozo MV, Guastalli EAL, Kariyawasam S, DebRoy C, Ávila FA. (2017 Jan). Captive and free-living urban pigeons (*Columba livia*) from Brazil as carriers of multidrug-resistant pathogenic *Escherichia coli*. Vet J.

[12] Cunha MPV, Oliveira MCV, Oliveira MGX, Menão MC, Knöbl T. CTX-M-producing *Escherichia coli* Isolated from urban pigeons (*Columba livia domestica*) in Brazil. J Infect Dev Ctries. 2019 Nov 30;13(11):1052-1056. doi: 10.3855/jidc.11441. 10.3855/jidc.11441 32087078

[13] Desvars-Larrive A, Ruppitsch W, Lepuschitz S, Szostak MP, Spergser J, Feßler AT, Schwarz S, Monecke S, Ehricht R, Walzer C, Loncaric I (2019 Aug). Urban brown rats (*Rattus norvegicus*) as possible source of multidrug-resistant Enterobacteriaceae and meticillin-resistant *Staphylococcus* spp., Vienna, Austria, 2016 and 2017. Euro Surveill.

[14] Guenther S, Bethe A, Fruth A, Semmler T, Ulrich RG, Wieler LH, Ewers C (2012). Frequent combination of antimicrobial multiresistance and extraintestinal pathogenicity in *Escherichia coli* isolates from urban rats (*Rattus norvegicus*) in Berlin, Germany. PLoS One.

[15] Semmler T, Stubbe A, Stubbe M, Batsajkhan N, Glupczynski Y, Wieler LH, Ewers C (2012). Comparable high rates of extended-spectrum-beta-lactamase-producing *Escherichia coli* in birds of prey from Germany and Mongolia. PLoS One.

[16] Hasan B, Islam K, Ahsan M, Hossain Z, Rashid M, Talukder B, Ahmed KU, Olsen B, Abul Kashem M. Fecal carriage of multi-drug resistant and extended spectrum β-lactamases producing *E. coli* in household pigeons, Bangladesh. Vet Microbiol. 2014 Jan 10;168(1):221-4. doi: 10.1016/j.vetmic.2013.09.033. 10.1016/j.vetmic.2013.09.033 24290770

[17] Mohsin M., Raza S., Schaufler K., Roschanski N., Sarwar F., Semmler T., Schierack P., Guenther S. (2017). High prevalence of CTX-M-15-type ESBL-producing *E. coli* from migratory avian species in Pakistan. Front. Microbiol..

[18] Mbehang Nguema PP, Onanga R, Ndong Atome GR, Obague Mbeang JC, Mabika Mabika A, Yaro M, Lounnas M, Dumont Y, Zohra ZF, Godreuil S, Bretagnolle F. Characterization of ESBL-Producing Enterobacteria from fruit bats in an unprotected area of Makokou, Gabon. Microorganisms. 2020 Jan 19;8(1):138. doi: 10.3390/microorganisms8010138. 10.3390/microorganisms8010138 PMC7022737 31963801

[19] Thomazelli L.M., Sinhorini J.A., Oliveira D.B.L., Knöbl T., Bosqueiro T.C.M., Sano E. (2021). An outbreak in pigeons caused by the subgenotype VI.2.1.2 of Newcastle disease virus in Brazil. Viruses.

[20] Topić Popović N., Kazazić S.P., Bojanić K., Strunjak-Perović I., Čož-Rakovac R. (2021). Sample preparation and culture condition effects on MALDI-TOF MS identification of bacteria: a review. Mass Spectrom. Rev..

[21] CLSI (2021). Clinical and Laboratory Standards Institute (CLSI). Performance Standards for Antimicrobial Susceptibility Testing. 31th ed. CLSI supplement M100.

[22] CLSI (2013).

[23] Magiorakos A.P., Srinivasan A., Carey R.B., Carmeli Y., Falagas M.E., Giske C.G. (2012). Multidrug-resistant, extensively drug-resistant and pandrug-resistant bacteria: an international expert proposal for interim standard definitions for acquired resistance. Clin. Microbiol. Infect..

[24] Manges A.R., Geum H.M., Guo A., Edens T.J., Fibke C.D., Pitout J.D.D. (2019). Global extraintestinal pathogenic *Escherichia coli* (ExPEC) lineages. Clin. Microbiol. Rev..

[25] Oteo J., Diestra K., Juan C., Bautista V., Novais Â., Pérez-Vázquez M. (2009). Extended-spectrum β-lactamase-producing *Escherichia coli* in Spain belong to a large variety of multilocus sequence typing types, including ST10 complex/a, ST23 complex/a and ST131/B2. Int. J. Antimicrob. Agents.

[26] Fuga B., Sellera F.P., Cerdeira L., Esposito F., Cardoso B., Fontana H. (2022). WHO critical priority *Escherichia coli* as one health challenge for a post-pandemic scenario: genomic surveillance and analysis of current trends in Brazil. Microbiol. Spectr..

[27] Palmeira J.D., Haenni M., Metayer V., Madec J.Y., Ferreira H.M.N. (2020). Epidemic spread of IncI1/pST113 plasmid carrying the extended-spectrum beta-lactamase (ESBL) *bla*_CTX-M-8_ gene in *Escherichia coli* of Brazilian cattle. Vet. Microbiol..

[28] Aworh M.K., Kwaga J.K.P., Hendriksen R.S., Okolocha E.C., Thakur S. (2021). Genetic relatedness of multidrug resistant *Escherichia coli* isolated from humans, chickens and poultry environments. Antimicrob. Resist. Infect. Control.

[29] Foster-Nyarko E., Alikhan N.-F., Ravi A., Thomson N.M., Kwambana-Adams B.A., Secka A. (2020).

[30] Maluta R.P., Logue C.M., Casas M., Meng T., Guastalli E. (2014). Overlapped sequence types (STs) and serogroups of avian pathogenic (APEC) and human extra-intestinal pathogenic (ExPEC) *Escherichia coli* isolated in Brazil. PLoS One.

[31] Fernandes M.R., Sellera F.P., Moura Q., Esposito F., Sabino C.P., Lincopan N. (2020). Identification and genomic features of halotolerant extended-spectrum-β-lactamase (CTX-M)-producing *Escherichia coli* in urban-impacted coastal waters, Southeast Brazil. Mar. Pollut. Bull..

[32] Lima L.S., Proietti-Junior A.A., Rodrigues Y.C., da Silva Vieira M.C., Lima L.N.G.C., de Oliveira Souza C. (2022). High genetic diversity and antimicrobial resistance in *Escherichia coli* highlight *Arapaima gigas* (Pisces: Arapaimidae) as a reservoir of quinolone-resistant strains in Brazilian Amazon Rivers. Microorganisms.

[33] Mohsin M., Hassan B., Martins W.M.B.S., Li R., Abdullah S., Sands K., Walsh T.R. (2021). Emergence of plasmid-mediated tigecycline resistance tet(X4) gene in *Escherichia coli* isolated from poultry, food and the environment in South Asia. Sci. Total Environ..

[34] Silva K.C., Moreno M., Cabrera C., Spira B., Cerdeira L., Lincopan N. (2016). First characterization of CTX-M-15-producing *Escherichia coli* strains belonging to sequence type (ST) 410, ST224, and ST1284 from commercial swine in South America. Antimicrob. Agents Chemother..

[35] Skarżyńska M., Zaja̧c M., Bomba A., Bocian Ł., Kozdruń W., Polak M. (2021). Antimicrobial resistance glides in the sky—free-living birds as a reservoir of resistant *Escherichia coli* with zoonotic potential. Front. Microbiol..

[36] Velhner M., Todorović D., Novović K., Jovčić B., Lazić G., Kojić M. (2021). Characterization of antibiotic resistance in *Escherichia coli* isolates from black-headed gulls (*Larus ridibundus*) present in the city of Novi Sad, Serbia. Vet. Res. Commun..

[37] Cao X., Zhang Z., Shen H., Ning M., Chen J., Wei H. (2014). Genotypic characteristics of multidrug-resistant *Escherichia coli* isolates associated with urinary tract infections. APMIS.

[38] Silva M.M., Sellera F.P., Fernandes M.R., Moura Q., Garino F., Azevedo S.S. (2018). Genomic features of a highly virulent, ceftiofur-resistant, CTX-M-8-producing *Escherichia coli* ST224 causing fatal infection in a domestic cat. J. Glob. Antimicrob. Resist..

[39] Nesporova K., Wyrsch E.R., Valcek A., Bitar I., Chaw K., Harris P. (2020). *Escherichia coli* sequence type 457 is an emerging extended-spectrum-β-lactam-resistant lineage with reservoirs in wildlife and food-producing animals. Antimicrob. Agents Chemother..

[40] Sellera F.P., Fernandes M.R., Moura Q., Lopes R.B., Souza T.A., Cerdeira L. (2018). Draft genome sequence of a *bla*_CMY-2_/IncI1-harbouring *Escherichia coli* D:ST457 isolated from coastal benthic organisms. J. Glob. Antimicrob. Resist..

[41] Wang M.-G., Fang C., Liu K.-D., Wang L.-L., Sun R.-Y., Zhang R.-M. (2022). Transmission and molecular characteristics of *bla*_NDM_-producing *Escherichia coli* between companion animals and their healthcare providers in Guangzhou, China. J. Antimicrob. Chemother..

[42] Zhong Y.M., Liu W.E., Zheng Z.F. (2019). Epidemiology and molecular characterization of *mcr-1* in *Escherichia coli* recovered from patients with bloodstream infections in Changsha, Central China. Infect. Drug Resist..

[43] Sampaio JL, Gales AC (2016). Antimicrobial resistance in Enterobacteriaceae in Brazil: focus on β-lactams and polymyxins. Braz J Microbiol.

[44] Rocha FR, Pinto VP, Barbosa FC (2016 Jun). The spread of CTX-M-Type Extended-Spectrum β-Lactamases in Brazil: A Systematic Review. Microb Drug Resist.

[45] de Souza da-Silva AP, de Sousa VS, de Araújo Longo LG, Caldera S, Baltazar ICL, Bonelli RR, Santoro-Lopes G, Riley LW, Moreira BM (2020 Nov). Prevalence of fluoroquinolone-resistant and broad-spectrum cephalosporin-resistant community-acquired urinary tract infections in Rio de Janeiro: Impact of *Escherichia coli* genotypes ST69 and ST131. Infect Genet Evol.

[46] Ferreira JC, Penha Filho RA, Andrade LN, Berchieri A, Darini AL (2014 Dec). IncI1/ST113 and IncI1/ST114 conjugative plasmids carrying blaCTX-M-8 in *Escherichia coli* isolated from poultry in Brazil. Diagn Microbiol Infect Dis.

[47] Botelho LA, Kraychete GB, Costa e Silva JL, Regis DV, Picão RC, Moreira BM, Bonelli RR (2015 Apr). Widespread distribution of CTX-M and plasmid-mediated AmpC β-lactamases in *Escherichia coli* from Brazilian chicken meat. Mem Inst Oswaldo Cruz.

[48] Botelho LAB, Kraychete GB, Rocha PB, da-Silva APS, Picão RC, Moreira BM, Bonelli RR (2020 Jan). CTX-M- and pAmpC-encoding genes are associated with similar mobile genetic elements in *Escherichia coli* isolated from different brands of Brazilian chicken meat. Microb Drug Resist.

